# Cultural adaptation and validity of the Malay version of the brief psychiatric rating scale (BPRS-M) among patients with schizophrenia in a psychiatric clinic

**DOI:** 10.1186/s12888-017-1553-2

**Published:** 2017-12-02

**Authors:** Anne Yee, Boon Seng Ng, Helenna Maria Hisham Hashim, Mahmoud Danaee, Huai Heng Loh

**Affiliations:** 1 Department of Psychological Medicine, University of Malaya Center for Addiction Sciences (UMCAS), Faculty of Medicine, University of Malaya, 50603 Kuala Lumpur, Malaysia; 2 Academic Development Centre (ADec), Faculty of Languages and Linguistics Building, University of Malaya, 50603 Kuala Lumpur, Malaysia; 3 Department of Medicine, Faculty of Medicine, UNIMAS, Kuala Lumpur, Malaysia

**Keywords:** Brief psychiatric rating scale, BPRS, Schizophrenia, Dimensionality reliability, Validity

## Abstract

**Background:**

This study evaluates the psychometric properties of the Malay version of the Brief Psychiatric Rating Scale (BPRS-M) among patients with schizophrenia in a psychiatric outpatient clinic.

**Methods:**

Ninety-nine schizophrenia outpatients were administered the Malay version of the Brief Psychiatric Rating Scale (BPRS-M), Malay version of Positive and Negative Syndrome Scale (PANSS), Malay version of Calgary Depression Scale for Schizophrenia (CDSS) and Malay version of World Health Organization Quality of Life – Brief Version (WHOQOL-BREF).

**Results:**

An exploratory factor analysis (EFA) of BPRS-M produced a seven-factor solution which accounted for 71.4% of the total variance. It exhibited fair internal consistency (Cronbach’s alpha coefficient of 0.75). “Positive symptoms” and “Resistance” factors had association with unemployment and number of antipsychotics, positively correlated with PANSS but negatively correlated with WHOQOL-BREF. “Mood disturbance” factor correlated with lifetime history of suicide attempts, Malay version of CDSS and WHOQOL-BREF (psychological). Both “Negative symptoms” and “Activation” factors were associated with male, lower education, unemployment and positively correlated with Malay version of PANSS but negatively correlated with WHOQOL-BREF.

**Conclusions:**

The BPRS-M demonstrated promising psychometric properties in terms of dimensionality, reliability, and validity that generally justifies its use in routine clinical practice in Malaysia.

## Background

The Brief Psychiatric Rating Scale (BPRS) [1] was developed to provide a highly efficient, rapid evaluation technique to assess treatment change in psychiatric patients, while at the same time yield a rather comprehensive description of major symptom characteristics. The present version of the instrument contains 18 items which assess the following symptoms: somatic concern, anxiety, emotional withdrawal, conceptual disorganization, guilt feelings, tension, mannerisms and posturing, grandiosity, depressive mood, hostility, suspiciousness, hallucinatory behavior, motor retardation, uncooperativeness, unusual thought content, blunted affect, excitement, and disorientation. The items are administered by a clinician based on an 8-point Likert scale (0 to 7) with total scores ranging from 0 to 126, with the higher scores representing greater severity of symptoms. Although BPRS has been expanded to 24 items (BPRS-E), many of the clinicians are still much familiar with BPRS 18 items in Malaysia.

This BPRS 18 has been extensively studied and has been proved to be a valid and reliable instrument in many languages such as Dutch, German, Portuguese [2–4]. Although its psychometric properties in terms of reliability, validity and sensitivity have been extensively examined, various factor solutions have been found due to the heterogeneity of the psychiatric diseases studied [5]. Nevertheless, four-, five- or six- factor solutions are frequently reported in schizophrenia samples [5]. The often reported factors are “Positive Symptoms” factor which is defined by items such as suspiciousness, unusual thought content, hallucinations, and bizarre behavior; “Anxiety-Depression” factor which is loaded with items such as depressed mood, guilt, tension and anxiety; “Activation” factor which overlaps with symptoms indicative for mania such as elevated mood, grandiosity and excitement; A “Negative symptoms or Anergia” factor that consists of a cluster of diminished abilities indicative of psychosis such as emotional withdrawal, blunted affect and motor retardation; A “Resistance” factor consists of uncooperative behaviors such as hostility, uncooperativeness; A “Disorganization” factor which has items that sometimes overlaps with “Positive symptoms” such as conceptual disorganization, bizarre behavior and disorientation. Occasionally, “Somatization” factor which consists of somatic concern and mannerisms and posturing is also found [5]. The previous study found that “Activation” factor was much better defined in the schizophrenic samples, and less defined in the mixed psychiatric samples [5].

The heterogeneity of schizophrenia psychopathology not only has led to different factors but also associated with broad features of disease, for examples age, gender, family history of psychiatric illness, clinical outcome, educational achievement and quality of life [6]. In the previous studies, “Negative Symptoms” factor is related to poor quality of life [6, 7] and poor educational achievement [8], “Positive symptoms” factor is associated with male, poor clinical outcome and poor quality of life [7], factor “Anxiety-Depression” is associated with higher rick of suicidal attempts [9], and the “Disorganization” factor is associated with family history of psychiatric illness [10].

Since the BPRS was developed to be a time-efficient, quick and an economical method of assessing treatment change among psychiatric patients, it would be beneficial for both clinicians and patients in Malaysia to have the BPRS validated in the Malay language (Malay version of BPRS, BPRS-M) as majority of the population here speaks this language. Furthermore, we are going to find out the relationship of the BPRS-M subscales to disease associated factors and quality of life in this study. Such an integrated analysis could help us to understand how the disease associated factors and quality of life relate to psychopathology, and this would be useful for the clinician to understand the impact of disease associated factors on clinical outcome [11].

Hence, the rationale for this study is first, to evaluate the validity and reliability of the Malay version of the Brief Psychiatric Rating Scale (BPRS-M) among patients with schizophrenia in a psychiatric clinic. Second, to assess the relationship between the BPRS-M subscale scores factors and sociodemographic, psychiatric history variables and quality of life.

## Method

### Study design

#### Stage 1

A forward translation of the Brief Psychiatric Rating Scale was conducted by two doctors who are bi-lingual in Malay and English. Later, the two forward translators and one member of the research group reviewed the two forward translations in order to create the “Reconciled Forward Translation”. The purpose for this translation was to ensure all demands of conceptual equivalence between the English version (“Source language”) and the Malay version (“Target language”) for each item were met.

### Stage 2

A back translation of the “Reconciled Forward Translation” was conducted by a local professional translator who was also bi-lingual in Malay and English. This production of backward version of BPRS was then compared with the original BPRS by a research team, which included a linguistic expert, psychiatrists, and a psychologist. All the misunderstandings, mistranslations or inaccuracies in the intermediary forward version of the questionnaire were corrected and thereby generating the “Final Forward Translation”. Later, this “Final Forward Translation” was pilot tested among 10 non-medical staffs from the University Malaya Medical Centre, who were native speakers of the Malay language. Any encountered difficulties with the items that were identified by these 10 respondents were resolved. The finalized version of BPRS in the Malay language (BPRS-M) was reviewed by a psychiatric consultant to ensure satisfactory face, semantic, criterion, conceptual and cultural equivalence.

### Stage 3

#### Population, setting and duration of study

The study population included all schizophrenic patients found in the psychiatric outpatient clinic of the University Malaya Medical Centre from August 2014 to July 2015. The study protocol was approved by the Medical Ethics Committee (MEC) of the University Malaya Medical Centre. The inclusion criteria were subjects who were: a) aged 18 years old and above, b) agreeable to participate in the study, c) diagnosed with schizophrenia by the first author who is a trained clinical psychiatrist by using the Diagnostic and Statistical Manual of Mental Disorders, 5th Edition (DSM 5) criteria, d) not having other major psychiatric illnesses, and e) able to understand English and Malay language. It was statistically appropriate to include at least 90 individuals with schizophrenia given that BPRS-M has 18 items, based on the subject-to-item ratio of 5:1 [12]. These patients had given their consent prior to the interview, and they were given the following questionnaires to complete – socio-demographic questionnaire, the BPRS and BPRS-M. Both the BPRS and BPRS-M were used to determine the parallel reliability.

### Instruments

#### Socio-demographic questionnaire

The socio-demographic questionnaire was used to record basic, yet relevant information about the participants of this study. The items listed were age, gender, ethnic group, marital status, education level, religion, and employment status. Participants were instructed to complete the questionnaire by filling in the blanks and selecting one response that best described them.

### Brief psychiatric rating scale (BPRS) and the Malay version of the Brief psychiatric rating scale (BPRS-M)

The present version of the instrument contains 18 items which assesses the following symptoms: somatic concern, anxiety, emotional withdrawal, conceptual disorganization, guilt feelings, tension, mannerisms and posturing, grandiosity, depressive mood, hostility, suspiciousness, hallucinatory behavior, motor retardation, uncooperativeness, unusual thought content, blunted affect, excitement, and disorientation. The items are administered by a clinician based on an 8-point Likert scale (0 to 7) with total scores ranging from 0 to 126, with the higher scores representing greater severity of symptoms. The Malay version of the BPRS is yet to be validated.

### Positive and negative syndrome scale (PANSS)

The scale was developed by Kay et al. (1987). It was found to be a reliable and valid tool to assess positive, negative and general psychopathologies in major psychiatric disorder, especially schizophrenia and other psychotic disorders [13]. It contained 30 items scale with positive, negative and general symptoms subscales. All 30 items are rated on a 7-point scale (1 = absent; 7 = extreme). The estimation of the reliability and internal consistency was high with the Cronbach’s alpha value of 0.809 and 0.931. In this study, The Malay version of PANSS (PANSS-Malay) was used.

### The Calgary depression scale for schizophrenia (CDSS)

This is a symptom scale for the assessment of depressive symptoms separate from positive, negative and extrapyramidal symptoms in patients with Schizophrenia [14, 15]. The scale was developed by Dr. Donald Addington in 1990. CDSS is a clinician rated, 9-item semi structured interview which assesses patient’s depressive symptoms over the past 2 weeks. Each item score represents depressive symptoms from 0 (absent) to 3 (severe). This scale has high internal consistency and significant strong correlations with other depression scale (Hamilton Depression Rating Scale, Beck Depression Inventory and Brief Psychiatric Rating Scale) [16]. In this study, The Malay version of CDSS (CDSS-Malay) was used.

### Quality of life assessment

Participants’ quality of life was assessed by using 26 items World Health Organization Quality of Life – Brief Version (WHOQOL-BREF). The Malay version of both WHOQOL-100 and WHOQOL-BREF has been validated [17, 18]. The Malay version of WHOQOL-BREF showed good internal consistency, test-retest reliability, discriminant validity and construct validity. Cronbach’s alpha value for the 4 domains ranged from 0.64 to 0.80, while the intra-class correlation coefficient ranged from 0.49 to 0.88 [17].

### Statistical analyses

All analyses were conducted using the Statistical Package for the Social Sciences version 21.0 (SPSS, Chicago, IL, USA). Descriptive statistics were computed for the baseline characteristics of the participants. First, the suitability of the BPRS-M data for factor analysis was verified by using the Bartlett’s test of sphericity and the Kaiser-Mayer-Olkin measure of sampling adequacy.

Construct validity was investigated by principal component analysis and varimax rotation. A factor loading of >0.40 was used to determine the items for each factor. The Cronbach’s alpha was used to assess the internal consistency of BPRS-M and subscale of BPRS-M. The normality of data was assessed using the skewness, kurtosis and boxplot methods [19]. Besides, PLS (partial least square) method using SMART-PLS 2 [20] was used for construct validity. Assessment of reflective measurement models includes some indicators such as composite reliability (CR), average variance extracted (AVE) and Cronbach’s alpha (α). In addition, the Fornell-Larcker criterion [21] and cross loadings were used to assess discriminant validity. The measurement model specifies the rules governing how the latent variables are measured in terms of the observed variables, and it describes the measurement properties of the observed variables. The measurement model is important as it provides a test for the reliability of the observed variables employed to measure the latent variables. A measurement model that offers a poor fit to the data suggests that at least some of the observed indicator variables are unreliable, and precludes the researcher from moving to the analysis of the structural model. Subsequently, analysis of variance or Pearson/spearman’s correlation coefficient was used as and when appropriate to examine the relationships between factor scores and sociodemographic, psychiatric history variables, BPRS, the Malay version of PANSS, the Malay version of CDSS and the Malay version of WHOQOL-BREF score. Following univariate analysis, multiple linear regression analysis was performed to identify the variables independently associated with each factor. Statistical significance was set at *p* < 0.05 as determined using two-sided tests.

## Results

A total of 99 patients were recruited. Majority of the participants were female (57.6%), Chinese (56.6%), single (72.7%) and unemployed (81.8%). Table 1 summarizes the patients’ characteristics.

**Table 1:** Sociodemographic characteristics and clinical features of all study subjects (*N* = 99)

Characteristic	N(%)
Gender	
Male	42 (42.4%)
Female	57 (57.6%)
Age (mean ± SD)	42.85 ± 11.77
Race	
Malay	14 (14.1%)
Chinese	56 (56.6%)
Indian	26 (26.3%)
Others	3 (3.0%)
Marital Status	
Single	72 (72.7%)
Married	27 (27.3%)
Education	
Primary	10 (10.1%)
Secondary	63 (63.6%)
Tertiary	26 (26.3%)
Occupation	
Unemployed	81 (81.8%)
Employed	18 (18.2%)
Duration of illness	
Less than 5 years	13 (13.1%)
5 years and above	86 (86.9%)
Number of hospital admission	
No admission	13 (13.1%)
1 – 2 admissions	45 (45.5%)
3 – 4 admissions	12 (12.1%)
5 and more admissions	29 (29.3%)
Family history of mental illness	34 (34.3%)
PANSS (mean ± SD)	
PANSS Positive	6.35 ± 4.75
PANSS Negative	16.77 ± 5.52
PANSS General	25.72 ± 6.74
PANSS Total	56.10 ± 14.32
CDSS (mean ± SD)	2.96 ± 4.05
WHOQOL-BREF score (mean ± SD)	
Physical	23.14 ± 3.09
Psychological	19.31 ± 3.52
Social	6.90 ± 2.64
Environment	24.20 ± 4.15
Overall	6.65 ± 1.50

*PANSS* Positive and Negative Symptoms for Schizophrenia, *CDSS* Calgary Depression Scale for Schizophrenia, *WHOQOL-BREF* World Health Organization Quality of Life – Brief Version

*SD* Standard Deviation, *N* number of patients

### Factor structure and internal consistency of BPRS-M

The Bartlett’s test of sphericity was significant (*p* < 0.01) and the Kaiser-Mayer-Olkin measure of sampling adequacy for the BPRS-M was 0.61 indicating mediocrity [22] and that factor analysis was appropriate. Seven factors were extracted with the Principle Component approach and the varimax rotation with Kaiser Normalization (eigenvalue >1.000) which accounted for 71.4% of the total variance. This result was consistent with the original BPRS. The seven factors which corresponded to the BPRS-M subscales referred to as “Positive Symptoms”, “Mood disturbance”, “Negative Symptoms”, “Activation”, “Resistance”, “Somatization” and “Orientation” (Table 2). However, “Orientation” subscale only consisted of one item.

**Table 2:** Factor analysis of Malay version of the Brief Psychiatric Rating Scale (BPRS-M)

Items	1 Positive Symptoms	2 Mood disturbance	3 Negative Symptoms	4 Activation	5 Resistance	6 Somatization	7 Orientation
4. Conceptual Disorganization	0.70						
11. Suspiciousness	0.85						
12. Hallucinatory Behavior	0.77						
15. Unusual Thought Content	0.69						
2. Anxiety		0.84					
5. Guilt Feelings		0.46					
6. Tension		0.81					
9. Depressive Mood		0.70					
3. Emotional Withdrawal			0.93				
13. Motor Retardation			0.50				
16. Blunted Affect			0.92				
8. Grandiosity				0.88			
17. Excitement				0.90			
10. Hostility					0.86		
14. Uncooperativeness					0.81		
1.Somatic Concern						0.93	
7. Mannerisms and Posturing						0.76	
18. Disorientation							1.00
Eigenvalue	3.95	2.16	1.64	1.52	1.38	1.20	1.00
Variance (%)	21.92	12.00	9.10	8.45	7.68	6.66	5.57

BPRS-M = Malay version of the Brief Psychiatric Rating Scale

Extraction Method: Principal Component Analysis. Rotation Method: Varimax with Kaiser Normalization. a. Rotation converged in 6 iterations. Loadings below 0.40 are suppressed

The BPRS-M exhibited fair internal consistency, with a Cronbach’s alpha coefficient for the total scale was 0.75, and those for other subscales were 0.78, 0.68, 0.73, 0.74, 0.55 and 0.63 respectively. All items had corrected-item total correlations of more than 0.70. Removal of items, if any, would not increase the Cronbach’s alpha coefficient.

The measurement model results for BPRS-M showed that all items had an outer loading above 0.5 which were above the threshold. These results revealed that critical ratio (CR) was 0.84 to 1. In addition, in this study, Average Variance Extracted (AVE) for all the subscales was above 0.5 (Table 4). Cronbach’s alpha, which provides an estimate of the reliability based on the inter-correlations of the observed indicator variables also was more than threshold (0.5). Thus, the results proved that convergent validity and construct reliability existed for the constructs of this study.

Discriminant validity is the extent to which a construct is truly distinct from other constructs by empirical standards. Thus, establishing discriminant validity implies that a construct is unique and captures phenomena not represented by other constructs in the model. Discriminant validity can be tested by examining the AVE for each construct against squared correlations (shared variance) between the construct and all other constructs in the model. A construct will have adequate discriminant validity if the AVE exceeds the squared correlation among the constructs [21, 23]. Based on Table 3, AVE for each construct is more than each of the squared correlation between constructs. Therefore, discriminant validity is adequate for all the constructs. As shown Table 4, the correlations between the latent variables ranged from −0.02 to 0.33, which were below the threshold 0.8, the squared correlations were less than the square root of the AVE by the indicators, demonstrating good discriminant validity between these factors [24].

**Table 3:** Results Summary for Measurement Model of Malay version of the Brief Psychiatric Rating Scale (BPRS-M) (Convergent Validity)

	AVE	CR	Cronbach’s Alpha
Positive Symptoms	0.57	0.84	0.79
Mood disturbance	0.52	0.81	0.70
Negative Symptoms	0.65	0.84	0.72
Activation	0.79	0.88	0.74
Resistance	0.69	0.82	0.56
Somatization	0.72	0.83	0.63
Orientation	1.00	1.00	1.00

*AVE* average variance extracted, *CR* composite reliability

**Table 4:** Correlation of latent variables and discriminant Validity of Malay version of the Brief Psychiatric Rating Scale (BPRS-M)

	Positive Symptoms	Mood disturbance	Negative Symptoms	Activation	Resistance	Somatization	Orientation
Positive Symptoms	**0.76**						
Mood disturbance	0.33	**0.72**					
Negative Symptoms	0.31	0.15	**0.81**				
Activation	−0.21	−0.16	−0.23	**0.89**			
Resistance	0.24	0.28	0.21	−0.11	**0.83**		
Somatization	0.08	0.20	0.08	−0.08	0.18	**0.85**	
Orientation	0.12	−0.04	−0.04	−0.02	0.10	−0.04	**1.00**

Bold number = square root of the average variance extracted (AVE)

The distribution of factor scores was approximately normal for total BPRS-M, “Positive Symptoms”, “Mood disturbance”, “Negative Symptoms” and “Activation” factor. However, “Resistance” (Skewness = 2.20, Kurtosis = 4.93), “Somatization” (Skewness = 2.93, Kurtosis = 8.30), “Orientation” (Skewness = 9.95, Kurtosis = 99.0) factors were not distributed normally.

As detailed in Table 5 and Table 6, no significant associations were found between factor scores and patient’s age, marital status, race, family history of mental illness, duration of mental illness and number of previous hospitalizations. Factor “Positive symptoms” was found to be associated with unemployment, number of antipsychotics, all the Malay version of PANSS and the Malay version of WHOQOL-BREF domain. Factor “Mood disturbance” displayed a positive correlation with lifetime history of suicide attempts, the Malay version of PANSS (positive, general and total), the Malay version of CDSS and the Malay version of WHOQOL-BREF (psychological). Factor “negative symptoms” was found to be associated with male, lower education, unemployment, all the Malay version of PANSS and the Malay version of WHOQOL-BREF domain. Factor “activation” displayed a correlation with lower education, unemployment, all the Malay version of PANSS and the Malay version of WHOQOL-BREF domain. Factor “resistance” displayed a positive correlation with number of antipsychotics, all the Malay version of PANSS and the Malay version of WHOQOL-BREF domain. Factor “Somatization” was found to have positive correlation with the Malay version of PANSS-total and general domains only.

**Table 5:** Comparison between Malay version of the Brief Psychiatric Rating Scale (BPRS-M) factor scores and sociodemographic and psychiatric history variables

	BPRS-M Factor scores							
	Positive Symptoms ^a^	Mood disturbance ^a^	Negative Symptoms ^a^	Activation ^a^	Resistance ^b^	Somatization ^b^	Orientation ^b^	Total ^a^
Gender	*t* = −0.73	*t* = 1.67	*t* = −2.56	*t* = −1.74	Z = −0.71	Z = −1.02	Z = −0.86	t = −0.73
Male	6.76(4.84)	2.83(2.88)	5.83 (3.03)	2.69(1.20) *	0.74(51.99)	0.52 (52.14)	0	16.69(8.09)
Female	6.05(4.70)	3.84(3.03)	4.47(2.26)	2.26(1.22)	0.74 (48.54)	0.26 (48.42)	0.02(50.37)	15.49(8.06)
Ethnicity	*t* = 0.48	*t* = −0.96	*t* = 1.22	*t* = 1.05	Z = −0.16	Z = −0.40	Z = −1.63	*t* = 0.30
Chinese	6.55(4.72)	3.16(2.92)	5.34(2.97)	2.55(1.35)	0.75(49.66)	0.30(49.38)	0.02(49.50)	16.21(8.17)
non- Chinese	6.09(4.82)	3.74(3.09)	4.67(2.23)	2.30(1.04)	0.72(50.44)	0.47(50.81)	0	15.72(8.00)
Marital Status	t = −0.96	*t* = −0.33	*t* = −0.10	*t* = 0.53	Z = −0.09	Z = −0.57	Z = −1.63	*t* = −0.99
Married	6.15(4.49)	3.22(2.87)	5.15 (1.92)	2.40(1.33)	0.63 (49.65)	0.37(48.33)	0	15.48(7.51)
non-married	6.43(4.87)	3.49(3.06)	5.01(2.93)	2.56(0.89)	0.78(50.13)	0.38(50.63)	0.01(51.33)	16.19(8.29)
Education	*t* = −1.96	*t* = −0.90	*t* = −4.61	*t* = −3.46	Z = −1.74	Z = −0.08	Z = −0.59	*t* = −3.11
≤ Secondary	6.90(4.65)	3.58(3.02)	5.73(2.43) **	2.68(1.07) **	0.84(52.51)	0.41(50.08)	0.01(50.18)	17.44(7.74)**
≥ Tertiary	4.81(4.77)	2.96(2.95)	3.15(2.49)	1.77(1.39)	0.46(42.96)	0.27(49.77)	0	11.96(7.67)
Employed	*t* = −2.38	*t* = 0.83	*t* = −1.86	*t* = −2.17	Z = −1.15	Z = −0.54	Z = −0.47	*t* = −2.04
Yes	4.00(4.17) *	3.94(3.02)	4.00 (2.17) *	1.89(0.90) *	0.44(44.11)	0.28(47.94)	0	12.56(7.96)*
No	6.88(4.73)	3.30(3.00)	5.28(2.74)	2.57(1.25)	0.80(51.31)	0.40(50.46)	0.01(49.50)	16.77(7.92)
Family history of mental illness	*t* = 1.30	*t* = −0.15	*t* = 0.10	*t* = −0.36	Z = −0.60	Z = −0.99	Z = −1.38	*t* = 0.87
Yes	7.21(4.29)	3.35(2.92)	5.09(2.94)	2.38(1.28)	0.85(52.00)	0.41()52.46	0.03 (50.96)	16.97(7.15)
No	5.91(4.94)	3.45(3.06)	5.03(2.57)	2.48(1.20)	0.68(48.95)	0.35()48.72	0	15.49(8.50)

Significant when *p* < 0.05*, *p* < 0.01**

^a^mean (standard deviation), Independent t-test

^b^mean (mean rank), Mann-Whitney test

**Table 6:** Correlation between Malay version of the Brief Psychiatric Rating Scale (BPRS-M) factor scores and age, psychiatric history variables, BPRS, PANSS, CDSS and WHOQOL-BREF score

	BPRS-M Factor scores							
	Positive Symptoms ^c^	Mood disturbance^c^	Negative Symptoms ^c^	Activation ^c^	Resistance ^d^	Somatic ^d^	Orientation ^b^	Total ^c^
Age	*r* = −0.11	*r* = −0.09	*r* = 0.02	*r* = 0.07	r = 0.02	*r* = −0.17	*r* = 0.21	*r* = −0.12
Duration of illness	*r* = 0.04	*r* = −0.07	*r* = 0.14	*r* = 0.12	*r* = 0.09	*r* = 0.16	*r* = 0.03	*r* = 0.03
Number of hospital admission	*r* = 0.10	*r* = 0.03	*r* = −0.05	*r* = −0.08	*r* = 0.05	*r* = −0.03	*r* = 0.14	*r* = 0.06
Number of antipsychotics	*r* = 0.24*	*r* = 0.11	*r* = −0.03	*r* = −0.10	*r* = 0.26**	*r* = −0.01	*r* = −0.08	*r* = 0.18
Total BPRS English								*r* = 0.95**
PANSS								
Positive	*r* = 1.00**	*r* = 0.24*	*r* = 0.39**	*r* = 0.32**	*r* = 0.25*	*r* = 0.14	*r* = 0.14	*r* = 0.83**
Negative	*r* = 0.58**	*r* = 0.11	*r* = 0.77**	*r* = 0.71**	*r* = 0.26**	*r* = 0.11	*r* = 0.08	*r* = 0.64**
General	*r* = 0.61**	*r* = 0.47**	*r* = 0.60**	*r* = 0.54*^*^	*r* = 0.35**	*r* = 0.24*	*r* = 0.05	*r* = 0.80**
Total	*r* = 0.76**	*r* = 0.36**	*r* = 0.68**	*r* = 0.61*^*^	*r* = 0.35**	*r* = 0.22*	*r* = 0.05	*r* = 0.86**
CDSS	*r* = 0.16	*r* = 0.72**	*r* = 0.07	*r* = 0.08	*r* = 0.16	*r* = 0.01	*r* = 0.04	*r* = 0.39**
WHOQOL-BREF								
Physical	*r* = −0.33**	*r* = −0.17	*r* = −0.30**	*r* = −0.26**	*r* = −0.23*	*r* = 0.11	*r* = −0.13	*r* = −0.37**
Psychological	*r* = −0.35**	*r* = −0.28**	*r* = −0.35**	*r* = −0.26**	*r* = −0.19	*r* = −0.13	*r* = −0.09	*r* = −0.46**
Social	*r* = −0.37**	*r* = −0.03	*r* = −0.58**	*r* = −0.50**	*r* = −0.11	*r* = −0.10	*r* = −0.02	*r* = −0.45**
Environment	*r* = −0.44**	*r* = −0.07	*r* = −0.47**	*r* = −0.39**	*r* = −0.12	*r* = −0.12	−0.05	*r* = − 0.47**
Overall	*r* = −0.27**	*r* = −0.11	*r* = −0.13	*r* = −0.14	*r* = −0.10	*r* = −0.10	0.02	*r* = −0.24*

*BPRS-M* Malay version of the Brief Psychiatric Rating Scale, *PANSS* Positive and Negative Syndrome Scale, *CDSS* Calgary Depression Scale for Schizophrenia, *WHOQOL-BREF* World Health Organization Quality of Life – Brief Version

Significant when *p* < 0.05*, *p* < 0.01**

^c^Pearson’s correlation coefficient

^d^Spearman’s correlation coefficient

To evaluate the effect of multiple variables, linear regression was used on the total BPRS-M score and subscales as dependent variables, while all variables found to be associated with one or more factors in univariate analyses (i.e., gender, occupational status, education, lifetime history of suicide attempts, number of previous admissions, the Malay version PANSS, the Malay version CDSS and the Malay version WHOQOL-BREF domains were entered as independent variables. In total BPRS-M score [*R* ^2^ = 0.85(adjusted 0.83), F (11, 87) = 45.69, *p* < 0.001] the only significant predictor was PANSS-positive subscales (β = 0.72, *p* < 0.001). In factor “Positive symptoms” [*R* ^2^ = 0.40 (adjusted 0.38), F (1, 97) = 54.99, *p* < 0.001] the significant predictors were the Malay version of PANSS general subscale scores (β = 0.60, *p* < 0.001) and the Malay version of WHOQOL-BREF overall domain ((β = −0.18, *p* = 0.02). In factor “Mood disturbance” [R2 = 0.52 (adjusted 0.50), F (1, 95) = 34.27, *p* = 0.04] the significant predictors were the Malay version of CDSS (β = 0.53, *p* < 0.001), the Malay version of PANSS-negative (β = 0.43, *p* < 0.001) and PANSS general subscales (β = −0.20, p = 0.04). In factor “Negative” [R2 = 0.67 (adjusted 0.66), F (1, 95) = 150.62, p = 0.04] the significant predictors were the education level (β = −0.13, *p* = 0.04), the Malay version of PANSS-negative (β = 0.61, *p* < 0.001) and the Malay version of WHOQOL-BREF social domain (β = −0.24, *p* < 0.001). Total PANSS score (β = 0.60, *p* < 0.001) was the only significant predictor for factor “Resistance”.

## Discussion

The current study, to the best of our knowledge, is the first to present the cultural adaptation, validity and reliability of the 18-item Malay Version of the Brief Psychiatric Rating Scale (BPRS-M) in a sample of patients with schizophrenia. Our study indicated that BPRS-M has adequate psychometric characteristics for construct validity, discriminant validity, concurrent and predictive validity, as well as its internal consistency.

In this study, a seven-factor model best explains the factor structure of symptoms in schizophrenia on the BPRS-M. The first six factors which corresponded to the BPRS-M subscales referred to as “Positive Symptoms”, “Mood disturbance”, “Negative Symptoms”, “Activation”, “Resistance” and “Somatization” which were consistent with other studies [2, 25]. However, the disorientation item (item 18) did not load on “Positive symptoms” or “Negative symptoms” as previous studies [5], instead item18 was the only item in “Orientation” factor. According to Dingemans et al. [2], this could be due to the selection of the sample. As compared with other studies [3, 26], this study only recruited the schizophrenia patients from a single outpatient clinic. Besides, only participants who were able to understand both languages (English and Malay) and agreed to be interviewed for nearly 1 h in an outpatient clinic setting were recruited. Hence, this study included patients who were stable and not very acutely psychotic,with all of them correponding to only being “mildly-moderately ill” in PANSS total score for schizophrenia [27]. Therefore, most of the particpants were fully orientated and score zero in item 18. Although the profile of factor loadings in our study slightly varied from previous studies, most previous factor studies found the factors similar to those we have identified here [2, 28, 29].

The BPRS-M also displayed a good convergent validity with all the subscales was above 0.5 threshold and good discriminant validity between these factors. Besides, the BPRS-M also exhibited fair internal consistency, with a Cronbach’s alpha coefficient for the total scale of 0.75. which is much higher than the original BPRS (Cronbach’s alpha coefficient = 0.69) [30]. The total score of BPRS-M correlated with original total score of BPRS (*r* = 0.95) and achieved statistical significant results, proved that the parallel reliability between two scales was good. This showed that the validated BPRS-M has successfully reduce the comprehension barrier when patients attempt the questionnaire, thus increases its usefulness as an instrument in detecting severity of psychiatric symptoms in this group of patients. This will also help the clinicians understand patient’s perceptions of the severity of their own symptoms before they administer the most appropriate treatment for their psychiatric conditions.

In the context of multiple factors, the variables independently associated with psychopathology were male, lower education, unemployment, number of antipsychotics, lifetime history of suicide attempts. Besides, BPRS-M is positively correlated with Malay version of PANSS and CDSS but negatively correlated with the Malay version of WHOQOL-BREF. Factor “Positive symptoms” and “Resistance” was found to be associated with unemployment and number of antipsychotics, positively correlated with all the domains in the Malay version of PANSS and negatively correlated with the Malay version of WHOQOL-BREF domains. Patients with positive symptoms were given more different type of antipsychotics medication. Our study showed that the positive symptoms and hostility had directly affected the schizophrenia patients’ quality of life. This finding was consistent with other studies [8, 31]. Meanwhile, it is also not surprising that factor “Mood disturbance” displayed a positive correlation with lifetime history of suicide attempts, Malay version of CDSS and Malay version of WHOQOL-BREF (psychological). This factor included symptoms typically related to depression and anxiety. A very similar “Depression–Anxiety” dimension also emerged in previous factorial studies of the 18-item BPRS in patients with schizophrenia [9]. Both factors “Negative symptoms” and “Activation” were found to be associated with male, lower education, unemployment and positively correlated with all domains in the PANSS but negatively correlated with all the Malay version of WHOQOL-BREF domains. This was consistent with previous studies [8, 30]. Women with schizophrenia have less severe symptoms, are more likely to secure a job and better quality of life when compared to males with schizophrenia [8].

Based on our analyses, none of the demographic variables, except for education level, could predict the factors of BPRS-M. The lower education level, together with low score in the Malay version of WHOQOL-BREF social domain could predict the higher score in factor “negative” of BPRS-M, and that too was consistent with a previous study [8]. It is not surprising that PANSS score could predict the factors of BPRS-M as there are many items in PANSS which are very similar to that of the BPRS-M.

A few limitations of this study might have impeded the generalization of our findings. Firstly, the 18-item BPRS was only administered to patients who had agreed to participate in this study. Secondly, our samples were recruited from an outpatient clinic in a tertiary hospital using convenience sampling. Thirdly, given the cross-sectional nature of this study, we were unable to assess the predictive validity of the BPRS-M. Lastly, recruitment only patients who were able to understand English and Malay language could give rise to selection bias.

## Conclusion

Despite its limitations, the findings showed that the BPRS-M could be a valid and reliable clinical instrument to routinely monitor psychopathology of the schizophrenia patients in our local outpatient setting. In the future study, there will be a need to addressing the sensitivity of change of the BPRS-M, so that the clinicians can use that to monitor the progress of their patients with schizophrenia.

## Abbreviations

AVE: Average Variance Extracted
BPRS-M: Malay version of the Brief Psychiatric Rating Scale
CDSS: Calgary Depression Scale for Schizophrenia
CFA: Confirmatory Factor Analysis
CR: Critical Ratio
PANSS: Positive and Negative Symptoms of Schizophrenia
WHOQOL-BREF: World Health Organization Quality of Life – Brief Version
